# Comparison of Huggins Coefficients and Osmotic Second Virial Coefficients of Buffered Solutions of Monoclonal Antibodies

**DOI:** 10.3390/polym13040601

**Published:** 2021-02-17

**Authors:** Jai A. Pathak, Sean Nugent, Michael F. Bender, Christopher J. Roberts, Robin J. Curtis, Jack F. Douglas

**Affiliations:** 1 Vaccine Production Program (VPP), Vaccine Research Center (VRC), Formulation and Stabilization Sciences Department, National Institute of Allergy and Infectious Diseases (NIAID), National Institutes of Health (NIH), 9 W. Watkins Mill Rd., Gaithersburg, MD 20878, USA; jai.a.pathak@gmail.com (J.A.P.); sean.nugent@nih.gov (S.N.); michael.bender2@nih.gov (M.B.); 2 Colburn Laboratory, Department of Chemical and Biomolecular Engineering, University of Delaware, Newark, DE 19716, USA; cjr@udel.edu; 3 Department of Chemical Engineering and Analytical Science, University of Manchester, Oxford Road, Manchester M13 9PL, UK; R.Curtis@manchester.ac.uk; 4 Materials Science and Engineering Laboratory, National Institute of Standards and Technology, 100 Bureau Drive, Gaithersburg, MD 20899-8544, USA

**Keywords:** monoclonal antibody, viscosity, Huggins coefficient, intrinsic viscosity, hard spheres, adhesive hard spheres, flexible polymers, second virial coefficient, static light scattering

## Abstract

The Huggins coefficient *k_H_* is a well-known metric for quantifying the increase in solution viscosity arising from intermolecular interactions in relatively dilute macromolecular solutions, and there has been much interest in this solution property in connection with developing improved antibody therapeutics. While numerous *k_H_* measurements have been reported for select monoclonal antibodies (mAbs) solutions, there has been limited study of *k_H_* in terms of the fundamental molecular interactions that determine this property. In this paper, we compare measurements of the osmotic second virial coefficient *B*_22_, a common metric of intermolecular and interparticle interaction strength, to measurements of *k_H_* for model antibody solutions. This comparison is motivated by the seminal work of Russel for hard sphere particles having a short-range “sticky” interparticle interaction, and we also compare our data with known results for uncharged flexible polymers having variable excluded volume interactions because proteins are polypeptide chains. Our observations indicate that neither the adhesive hard sphere model, a common colloidal model of globular proteins, nor the familiar uncharged flexible polymer model, an excellent model of intrinsically disordered proteins, describes the dependence of *k_H_* of these antibodies on *B*_22_. Clearly, an improved understanding of protein and ion solvation by water as well as dipole–dipole and charge–dipole effects is required to understand the significance of *k_H_* from the standpoint of fundamental protein–protein interactions. Despite shortcomings in our theoretical understanding of *k_H_* for antibody solutions, this quantity provides a useful practical measure of the strength of interprotein interactions at elevated protein concentrations that is of direct significance for the development of antibody formulations that minimize the solution viscosity.

## 1. Introduction

Recombinant proteins such as immunoglobulins (IgGs) are now routinely administered to patients at relatively high protein concentrations, often exceeding 100 mg/mL. Biopharmaceutical development scientists are confronted with serious challenges, primarily centered around protein aggregation [1,2,3], general colloidal instability, and elevated viscosity, *η*, all of which influence product stability and can render parenteral administration difficult [4,5]. Meanwhile, protein therapeutics provide relatively high in vivo potency, due to their large molecular weights and high required doses on the order of 1–2 mg/kg of the patient body weight and the relatively low dose volumes (<1.5 mL) required for subcutaneous drug administration [6]. At these high protein concentrations, the protein drug may “degrade” through aggregation and high solution viscosity, which are common challenges that must be overcome [1,7,8]. Specifically, the aggregation of a protein biotherapeutic can lead to reduced drug product potency and a greatly increased immunogenic response upon administration to patients [9,10,11]. The current pressing need for these highly concentrated protein therapeutics drives research in this field, especially antibody drugs. Then, the development of metrologies for quantifying protein–protein interactions, and for anticipating changes in the viscosity of protein solutions with changes in protein concentration and other changes in processing and clinical conditions, is a topic of intense scientific research and practical technological interest.

A discussion of the concentration dependence of viscosity can be mathematically framed in a virial framework, starting with the intrinsic viscosity, [*η*]. Intrinsic viscosity is defined in the zero-protein concentration (*c*_2_) limit of the quotient of specific viscosity, *η_SP_*, and *c*_2_. Tanford demonstrated that [*η*] is an appropriate dilute solution viscometric descriptor [12], as in the case of synthetic polymer solutions [13],

(1) $$\left[\eta \right]=\underset{c_{2}\to 0}{Lim}\frac{\eta_{SP}}{c_{2}}=\underset{c_{2}\to 0}{Lim}\frac{\eta -\eta_{S}}{\eta_{S}c_{2}}$$

Below, *η_S_* and *η* denote buffer and protein solution viscosity, respectively. The relative viscosity of the protein solution, *η_R_*, can be defined as *η_R_* = *η*/*η_S_* and is directly related to the specific viscosity (*η_SP_* = *η_R_* − 1). The relative viscosity can also be expressed as a virial expansion power series in the solute (protein) concentration:

*η_R_* = 1 + [*η*] *c*_2_ + *k_H_* ([*η*] *c*_2_) ^2^ + …. (2)

The second hydrodynamic virial coefficient or “Huggins coefficient” *k_H_* is defined in the concentration regime where two-body interactions dominate, similar to the osmotic second virial coefficient, *B*_22_, which is obtained from osmotic pressure measurements. By providing a hydrodynamic measurement of inter-protein interactions, specifically the effect on the solution viscosity for IgG solutions beyond the dilute regime, *k_H_* may shed new insights into our understanding of crowded protein solution physics. The studies presented here explore the hypothesis that the hydrodynamic information provided by *k_H_*, as well as contributions to thermodynamic and conformational information, may be of significant value in characterizing protein solutions at elevated protein concentrations.

An in-depth examination of *k_H_* also offers an opportunity to scrutinize and evolve classical theories regarding the intrinsic physical nature of proteins. Recent work by the authors [14,15,16] has explored the hypothesis that proteins are better idealized as polymeric structures rather than colloidal hard spheres, which is a subject of great relevance to modeling the solution hydrodynamic and thermodynamic properties of these macromolecules. Negatively and positively charged patches are present on protein molecules, suggesting that these systems might be idealized as patchy colloidal particles [17] (We discuss the rationale of this simple protein model below). The colloidal depiction has often been assumed to be a reasonable initial model for relatively compact proteins in their native globular state where deviations from the spherical shape are relatively small compared to swollen linear polymers [18]. However, the invocation of a colloidal model of proteins should not preclude a consideration of models that incorporate a description of directional interactions and molecular structure and variations in the rigidity of these molecules, and we discuss some of these models below. The solution properties of monoclonal antibodies examined in this paper are compared to the classic hard sphere colloid and flexible polymer models as zero-order reference models with a view toward developing an adequate minimal model of these protein solutions.

Inter-protein interactions in the solution state can be generally divided into short-range “SR” attractive/adhesive interactions (hydrophobic, van der Waals, H-bonding, excluded volume, etc.) and long-range “LR” interactions, which are typically electrostatic in nature (monopole, dipole, quadrupole, etc.) (We discuss these interactions and their significance for the properties of antibody solutions in a separate section below.). Solution ionic strength (*I*) is a convenient experimental tool to turn LR electrostatics “on” at low *I* or “off” at high *I* by adding salt to screen electrostatic interactions through the Debye screening effect. The application of this model seems to be most suitable for the high *I* regime, where the electrostatic screening should be especially great, as postulated by Kastelic et al. [19]. With these assumptions in mind, we consider the adhesive or “sticky” sphere model of Russel [20], henceforth referred to as the “Russel model”, as a potentially suitable coarse-grained of protein/antibody solutions with appreciable salt and concentrations below the infinite dilute limit as well as below the concentrated solution regime. We emphasize that Russel’s simplified hydrodynamic model for *k_H_* neglects charge, dipole interactions, and protein conformation effects that are clearly important in protein solutions so that the applicability of this model, even in the high salt, remains a question that must currently be decided by measurement in the absence of any theory of sufficient generality to describe all the potential interactions that one might consider as being possibly relevant to the complex solutions. The Russel model is a “coarse-grained” model, which is a dignified way of saying that it involves radical approximations. Despite the limitations apparent in this type of model, it still provides a benchmark model against we may gauge the behavior of real protein solutions, and we may hope that the dimensionless reduced variables defined by this model might have a greater validity than the model itself, as in the case of van der Waals model of the equation of state of liquids.

Russel’s simple model of *k_H_* of particles having attractive interparticle interactions extends the classical calculation of Batchelor [21] to account for Brownian stress contributions to hard sphere interparticle interactions modeled by the classical Baxter sticky hard sphere model [22]. In this context, the “strength” of the attractive interaction between spherical particles with sticky interfacial interactions is defined by a phenomenological “stickiness parameter”, *τ*. Since the direct experimental measurement and the physical interpretation of the parameter *τ* is problematic, Douglas and coworkers [23] directly related *k_H_*to the second osmotic virial coefficient *B*_22_, which is a well-known experimental metric of interparticle interaction based on the same sticky sphere frameworks of both Baxter and Russel [20,22]. The second virial coefficient *B*_22_ within this model is defined as

(3) $$B_{22}=B_{22,ex}+\frac{1}{2}\int_{d_{p}}^{\infty }\left[1-e^{\left(-\beta w\left(r\right)\right)}\right]4\pi r^{2}dr$$

where “*ex*” denotes the purely repulsive excluded volume (hard sphere, *HS*) contribution. Note that for hard spheres, we have the simple geometric result, *B*_22,*HS*_ = *V_ex_*/2 = (2π/3) *d_p_*^3^, where *V_ex_*and *d_p_* denote excluded volume and sphere diameter. *β*^−1^ = *k_B_T* where *k_B_* and *T* denote Boltzmann’s constant and absolute temperature, respectively, and *w*(*r*) is the potential of mean force, i.e., the averaged force between a pair of molecules vs. radial separation, *r*.

With general equation of state ideas in mind as a potential framework for extending the application of this type of coarse-grained model to describe systems of interest in practical applications, *B*_22_ can be normalized by the steric contribution limit for mAbs to make this property dimensionless (*Ψ* ≡ *B*_22_*/B*_22_,*_ST_*). Then, this reduced virial coefficient *Ψ* can be used to enable a direct comparison of model predictions to measurements without any *adjustable parameters*. In particular, Douglas and coworkers [23] noted that Russel’s result for *k_H_* can be re-expressed in terms of *Ψ* as follows,

*k_H_* = 3.42 − 2.43 *Ψ* (4)

This expression is a natural extension of a previously derived linear relationship [24,25] between the leading order hydrodynamic virial *k_D_* coefficient for the collective diffusion coefficient *D_c_* and *Ψ*. This quantity likewise provides a basic measure of solution interparticle particle and is also currently being intensively investigated as a potential predictive measure of the stability of antibody solutions against aggregation [26,27].

Equation (4) highlights the fact that attractive interparticle interactions exist when the proteins are near their theta point at which *B*_22_ = 0 increases *k_H_* (*Ψ* = 0) by roughly a factor of 3 in comparison to the case of hard spheres modeling the case of “good solvent” where the interparticle interactions are purely repulsive interactions, i.e., *Ψ* = 1. We note that Batchelor‘s hard sphere result [21,28], *k_H_* (*Ψ* = 1) = 0.99 is recovered by Equation (4) so that Equation (4) is consistent with this classic colloid theory result. Many past studies have assumed spheres with a short range “sticky” attraction might provide a “universal” model of suspended particles systems with attractive interactions, including colloidal particles, polymers, and biological macromolecules such as proteins. Below, we also compare our results to another coarse-grained model of proteins that emphasizes that proteins are after all polymers of amino acids rather than spherical particles, and we compare to existing theoretical and measurement results for flexible polymers having a short-range attractive interaction defined in terms of *Ψ*, where the size is defined in terms of the polymer radius of gyration. Before proceeding to describe our experimental observations on model antibody solutions and compare our findings with the expectations of the sticky sphere and flexible chain models of proteins, we discuss the inherent limitations of these rather simplistic coarse-grained protein models and the qualitative physical origin of the interprotein interactions that make protein characterization highly challenging from both theoretical and technological viewpoints. The sticky sphere and flexible chain models of antibody proteins can only be considered stepping-stones to a more adequate theoretical framework of antibody solutions.

### Brief Overview of the Complexities of Protein–Protein Interactions and Protein Association

We emphasize at the outset that both the idealized colloidal sphere and polymer models of antibody protein solutions should be viewed with some skepticism and, indeed, our observations on antibody protein solutions below indicate that this skepticism is well-justified. Many authors have previously reported the tendency of antibody proteins to dynamically associate in solution, which is a phenomenon that directly impacts the shear viscosity and other properties of these solutions [6,8,29,30,31,32]. These increases in viscosity with increasing protein concentration depend on the temperature, protein concentration, buffer excipients, pH, and other general “solution conditions”.

We will discuss below observations indicating that there is a general tendency of proteins, as a class, to form supramolecular clusters [33,34,35,36,37,38], along with accompanying strong changes in the solution viscosity. This phenomenon cannot be understood from a simple colloid sphere model with a sticky interaction or neutral polymer model. Given the importance of this phenomenon for understanding the solution properties of antibody solutions, and protein solutions broadly, some explanation of the physical factors contributing to protein clustering seems warranted, and we devote this section to this matter.

While the biological community has long been concerned with a wide range of variables that complicate our understanding and prediction of inter-protein interactions [39,40,41,42], it is easy to identify the greatest shortcoming of the simple colloidal sphere and neutral flexible polymer models of proteins. Regardless of whether proteins are in the globular form of some globular proteins or take the form of swollen flexible polymer chains that are characteristic of intrinsically disordered proteins, protein molecules are most fundamental chains of amino acids, which are *dipolar molecules.* Moreover, protein molecules have macrodipoles, whose magnitudes tend to be especially large for proteins whose biophysical functionalities depend on their capacity to form strong molecular associations. In particular, dipole moments on the order of 1000 Debye (*D*) are rather common [43,44,45,46,47,48] in strongly associating proteins, while values on the order of 100 Debye are more typical of globular proteins whose function does not normally involve large scale supramolecular organization. Many proteins, including monoclonal antibodies, which bind to other “ligands” as part of their functional activity, lie in the middle range of this extremely wide range of dipole moment values, and we may expect this range to be representative of antibody proteins, since they also bind to receptor targets expressed on/by cells. Antibodies form strong ligand–receptor complexes as part of their bio-activity (potency), and they are also intermediate structurally between globular proteins and denatured proteins that structurally resemble random coil polymers.

Van Workum and Douglas [47,48] have argued that dipolar, and sometimes strong quadrupole interactions, as in the case of tubulin and other sheet-forming proteins, are the primary interactions governing protein self-assembly where hydration-related relatively short-range interactions also play an important role in stabilizing the supramolecularly-assembled linear and branched polymer chain structures, membrane-like sheets, and closed “nanotube” and shell-like or “capsid” structures that naturally form this delicate balance between long-range directional and short-range attractions [47,48]. These dipolar interactions have been shown to have significant implications for the phase stability of solutions of dipolar particles [49] and protein solutions, by extension. The recognition of the predominant importance of dipolar interactions in protein and polypeptide solutions is not new. Brant and Flory noted that each amino acid group should contribute about 3.5 *D* to the polypeptide macrodipole [50], and later, other researchers emphasized the importance of protein macrodipoles in stabilizing protein structure [51,52] by influencing ion association and disassociation. We also mention the highly relevant work of Kirkwood and Shumaker [53,54], Antosiewicz and Porschke [46], and the more recent work by Adzic and Podgornik [55], which all emphasize the importance of charge and dipole fluctuations in contributing to the observed effective macrodipole interactions of proteins. These works make it clear that knowledge of protein structure is not sufficient to specify the multipole interactions of proteins in solution, which can rather complicate theoretical modeling of these solutions. Minimally, the protein and ion association must be modeled to account for these charge fluctuation effects, which means that solvation effects must be accounted for in the modeling.

Based on these general considerations, it should come as no surprise that recent experimental studies of dipole–dipole interactions in antibody solutions have revealed a strong correlation between the antibody dipole moment, which can depend appreciably on pH, and the macroscopic viscosity of the antibody solution. This has led to a general recognition of the importance of these interactions [34,56], and this knowledge has also led to successful efforts at developing additives that modulate the dipolar interactions in order to inhibit the protein self-assembly process for clinical applications relating to antibody drug delivery [31]. This knowledge appears to offer a very promising conceptual framework for engineering improved antibody formulations.

Then, the measured viscosity of protein solutions depends on the number of proteins in these aggregates, their polydispersity and size, as well as whether there is a persistence of their aggregated state. We are clearly dealing with a very complex class of materials. As noted before, protein clustering is particularly natural in antibody protein solutions because of their prevalent dipolar interactions. This is the real problem that we are up against in our effort to establish a general metric for quantifying interparticle interactions strength in protein formulations. In previous work [14], we pointed out clear evidence for the inadequacy of modeling proteins as spherical particles with short-range sticky interactions, and we then suggested that we should address the inherently polymeric nature of proteins. The findings of the present work imply that we must consider multipole interactions or at least the directional aspect of such interactions in any minimal model of the protein solutions. Attempts to model such interactions are ongoing, and briefly, we outline some recent promising attempts to address this type of interaction.

Recent simulation studies have begun the ambitious task of incorporating such interactions [57] into molecular dynamics of protein solutions, but this effort requires the incorporation of explicit solvent to address ion and protein solvation phenomena [58] and will require large-scale simulations. Despite these complexities, we may gain some qualitative insight into protein assembly from the Stockmayer fluid [47], which is a minimal model of proteins in solution that combines a particle dipole–dipole interaction with a competitive van der Waals interaction, as described by the well-known Lennard–Jones interaction. When the dipole–dipole interaction strength is strong [47,48], the particles in this model exhibit a large degree of reversible self-assembly into linear polymer chains whose size depends on temperature, particle concentration, and other thermodynamic physical factors, as found in found in previous studies of amyloid protein self-assembly of amyloid fibrils [59,60,61,62] and actin polymerization under equilibrium conditions [63,64,65]. Exact analytical calculations of *B*_22_ of the associating particles in the Stockmayer model fully account for their reversible associations [47] through their renormalization of the second virial coefficient. Van Workum and Douglas [47,48] have explained why the Stockmayer model, which includes competitive van der Waals and dipolar interactions, and its quadrupolar interaction generalization, are highly attractive general coarse-grained models for protein self-assembly that address their strong dipolar and quadrupolar interactions [47,48], and they also describe their corresponding general tendency to self-assemble into dynamic polymeric structures. Then, we have some hope that aggregating particle systems exhibiting weak but reversible self-association might be described phenomenologically by this type of highly coarse-grained protein model that accounts for the highly directional interactions that are intrinsic to proteins and many other biological and synthetic supramolecular assembling molecules [66].

There are also some coarse-grained models that neglect the long-range nature of the multipole interactions for computational expediency. In particular, the long-range multipole interactions are replaced by “sticky” spots on the surface of a spherical particle modeling the protein, so we return again to what could be called a colloidal protein model. In particular, this type of highly coarse-grained protein model has been found to be useful in gaining insights into the phase separation of protein solutions [17,19,67,68,69,70]. Recent work has adapted this type of “spot model” to describe antibody proteins based on an extension that replaces the individual sphere with spots by small polymers of beads that have spots in which the polymers form a Y-like configuration (a star in polymer science parlance) that is characterized by the geometrical structure of real antibodies in solution. This is a somewhat more elaborate but still highly coarse-grained model of antibodies in solution that has allowed the quantitative estimation of the polymer cluster size distributions, the Huggins coefficient, and the second osmotic virial coefficient over a wide range of thermodynamic conditions [71]. This model, and the related model of Skar-Gislinge et al. [72], have not yet been shown to quantitively agree with measurements. This type of modeling is still a work in progress, but this approach seems very promising.

In another important development, the sticky spot model has been extended to include an explicit solvent and counterions [71]. An extension of the antibody model to incorporate such hydration-related physics would probably go a long way toward having a coarse-grained model of predictive value for predicting at least trends in antibody solution properties if the parameters in the model were carefully determined through a synergistic comparison between simulation and measurement efforts on protein solutions. Hofmeister specific ion effects associated with ion hydration are implicated in a wide range of biological phenomena, including protein binding and stability [58], and as general factor relevant to excipient additives [73] for antibody formulations to improve their solution [74,75]. The general lack of understanding of Hoffmeister and other protein and ion hydration effects is perhaps the weakest link in all existing methods aimed at modeling the properties of protein solutions, while at the same time, these effects offer great opportunities for practical applications for enhanced antibody solution stabilization and an enhanced knowledge of aqueous solutions broadly.

Another basic, and related, problem is that molecular flexibility [76,77], which is influenced by hydration, molecular structure, ion association, and many other factors, makes an often large contribution to the entropy of binding and thus the overall binding affinity [78,79]. The relation of antibody rigidity in the regulation of molecular binding strength has been especially studied in antibodies because their rigidification occurs rapidly in the course of the “maturation” of the binding affinity to the antigen’s final “evolved” value [80,81,82]. This same process seems to be involved for conserved proteins and their fundamental binding processes in the adaption of organisms to different environments [79]. A general lack of understanding of how rigidity influences molecular binding in aqueous solutions is a central problem that has often prevented the successful prediction of molecular binding constants of protein-based drugs [83,84,85].

There is ongoing work to develop an effective minimal model of antibody solutions that involves just enough “coarse-graining” of the protein solution physics, along with a measurement program to establish basic trends in the model parameters of such a theory to enable the organization and interpretation of the rapidly growing number of measurements in this critically important class of materials for human health. Such a model should be useful to the biopharmaceutical community in the rational design of stable antibody formulations optimized in terms of efficacy, safety, and economic costs for their intended clinical use.

While we are waiting for the development of an effective theory of the properties of antibody solutions, we feel that a focus on *B*_22_ and on corresponding properties greatly dependent on *B*_22_, such as *k_H_*, and the corresponding virial for the collective diffusion coefficient *k_D_* [26,27] might provide rational metrics for characterizing inter-protein interaction strength under conditions where near-equilibrium association and relatively good dispersion prevails. This ultimate goal transcends whether the protein data itself quantitively ‘fit’ the theoretical predictions of the coarse-grained model. Protein solutions exhibiting a high degree of irreversible association (“aggregation”) are excluded from our current consideration because of the highly negative immune response that such solutions would trigger upon administration to patients. Therefore, the systems studied have been “selected” to eliminate cases of irreversible protein aggregation.

## 2. Materials and Methods

Broadly neutralizing monoclonal antibodies “mAb-1” [86], “mAb-2” [87], and “mAb-3” [88] (Table 1), targeting the CD4-binding region of human immunodeficiency virus 1 (HIV-1), are in development by the National Institutes of Health (NIH) Vaccine Research Center (VRC) for clinical research. Their F_ab_ crystal structures and primary amino acid sequences are available in the Protein Data Bank (PDB). To retain their unique biological activities, the sequence of the mAbs were minimally optimized for stability. The antibodies were expressed by Chinese Hamster Ovary (CHO) cells, purified by protein A chromatography followed by a polishing step consisting of ion exchange resin chromatography, and then concentrated in their respective formulation buffer to 100 mg/mL nominal protein concentration (Table 1). All IgG solutions were stored at ≤ −65 °C, thawed immediately before use, and filtered via 0.22 µm polyvinylidene fluoride (PVDF) syringe filters prior to measurements.

For the pH-dependence studies, mAb-3 stock solution at 40 mg/mL was dialyzed against 20 mM histidine-acetate buffer at 4.2 ≤ pH ≤ 6.2, using Slide-A-Lyzer™ dialysis cassettes with a molecular weight cutoff of 20,000 (ThermoFisher Scientific, cat. # 66012, Waltham, MA, USA). Following dialysis, pH was confirmed, and samples were formulated with the required NaCl. All buffer components were compendial USP grade for high purity (VWR Avantor, Radnor, PA, USA).

### 2.1. Size Exclusion Chromatography (SEC)

Immediately prior to testing, mAb samples were diluted with 2X PBS to 1 mg/mL. Fifty μg of mAb was injected onto the Waters Acuity^TM^ BEH (ethylene bridged hybrid, Waters, Milford, MA, USA) SEC column (P/N: 186005225; 4.6 mm × 150 mm I.D, particle size: 1.7 μm, pore size: 20 nm) of the Waters Acuity UPLC H-Class system (Waters, Milford, MA, USA). An isocratic 2X PBS mobile phase (pH 7.4) was run at a flow rate of 0.4 mL/min for 6 min. UV absorbance was detected at 280 nm using Empower 3 software (Waters, Milford, MA, USA). The percentage monomer, aggregate, and fragment for each system was determined by integration of the area under the peak of UV signal vs. elution time. Only one SEC injection/measurement was performed for each sample, as is typically done.

### 2.2. Capillary Iso-Electric Focusing (cIEF)

Capillary Isoelectric Focusing utilizing the iCE3 platform (IEF, PrinCE micro injector auto sampler, iCE 3 software package v. 3.0 and Chrom Perfect iCE3analysis software v. 6.0.4; ProteinSimple, San Jose, CA, USA) is an assay used to evaluate the charge heterogeneity of charged isoforms of proteins. cIEF is a charge-based separation technique that separates molecules based on their isoelectric point (pI). Samples are prepared by mixing the protein of interest with selected carrier ampholytes and pI markers. Then, the samples are loaded into the capillary cartridge (cIEF cartridge FC-coated (ProteinSimple, San Jose, CA, USA, Cat # 101701). Acid and base are added to the electrolyte tanks on the cartridge, and a voltage is applied in which analytes are focused at their pI. The focusing step is captured in real time by a charge-coupled device (CCD) camera, which takes a picture of the entire length of the capillary column every 30 s. The resulting separation is an electropherogram that identifies the pI and absorbance of the protein peaks. The samples comprised 50 µL of 2 mg/mL mAb, 100 µL of master mix solution, 150 µL of ultra-pure water. Each single mL of master mix solution comprised 740 µL of 1% Methyl Cellulose Solution, 200 µL of 8–10.5 ampholyte, and 20 µL each of 3–10 ampholyte, pI standard 7.9, and pI standard 10.1. The focusing times in focus periods 1 and 2 were 3.0 and 10.0 min, respectively, while the voltages in focus periods 1 and 2 were 500 and 3000 V, respectively.

### 2.3. Viscometry

Samples were prepared from 100 mg/mL stock mAb solutions and diluted between 0.1 and 90 mg/mL in their respective buffers. Concentrations were confirmed by UV/Visible spectroscopy (Agilent 8453 UV-Visible spectrophotometer, Agilent Technologies, Santa Clara, CA, USA) using gravimetric dilutions.

Solution *η* of mAbs was measured using the Viscosizer (Malvern Panalytical, Malvern, UK). Measurements were performed at 25 °C using an uncoated fused silica capillary, with an inner diameter of 75 µm and 130 cm total length. Air-driven viscosity measurements were performed at a constant pressure differential of 3000 mbar, which translated to shear rates between 1300 and 4600 s^−1^. The measured viscosity was independent of shear rate for all solutions.

### 2.4. Osmotic Second Virial Coefficient Determination

Static light scattering (SLS) was used to determine the osmotic second virial coefficient (*B*_22_). Samples were prepared from 100 mg/mL stock solutions by gravimetric dilution to 2, 4, 6, 8, and 10 mg/mL with their respective buffers. SLS measurements were performed using a high-throughput fluorimeter/scattering system (UNcle, Unchained Labs, Pleasanton, CA, USA). Each measurement was conducted at 25 °C and consisted of 10 acquisitions of 30 s each, with laser power and attenuation set to 100% and 25% respectively. The excess Rayleigh scattering ratio, *Kc*_2_/*R_θ_*, was determined from 660 nm wavelength data. Employing classical Debye–Zimm analysis [89], the *B*_22_ values were determined from the slope of the Debye–Zimm plot. National Institute of Standard and Technology (NIST) mAb Reference Material 8671 (NIST, Gaithersburg, MD, USA) was used as an absolute reference standard for SLS to determine the light scattering constant *K*, which assumes that the refractive index increments (*dn*/*dc*) for each antibody solution are similar to each other.

### 2.5. Hydrated Protein Molecular Volume: Atomistic Monte Carlo Computer Simulations

Antibody molecular volumes, *v*_2_, were calculated by simulating the virial coefficient *B*_12_, which quantifies protein–water interactions; subscripts 1 and 2 denote water (solvent) and protein (solute), respectively. In the limit where only steric interactions (*ST*), i.e., excluded volume effects, are present, 2*B*_12,*ST*_ is an estimate of the excluded volume of protein with respect to a single water molecule approximated as a solid sphere of diameter 0.3 nm (the pre-factor 2 comes from the derivation in Equation (5), due to the ½ pre-factor). This is equivalent to the total volume of water that will be excluded from the protein molecule in solution. Since proteins are expected to always carry at least one solvation layer, due to the energetic penalty of disrupting such a layer, 2*B*_12,*ST*_ is a perfect estimate of the protein-excluded volume.

*B*_12,*ST*_ was computed using the Mayer Sampling with Overlap Sampling (MSOS) algorithm [90] for an all-atom description, including hydrogens, of a generic mAb with variable F_ab_ regions, taken using PDB F_ab_ and generic PDB 1IGT F_c_ (fraction crystallizable) crystal structures. The MSOS algorithm efficiently solves the equation of *B*_12_ for purely steric interactions [91,92]. The contribution from the antibody hinge regions is small, ≈ few %, based on the total number of amino acids there, and hence, it was neglected as an engineering approximation:

(5) $$B_{12}=-\frac{1}{2}\int \int \int_{r\Omega_{1}\Omega_{2}}\left[e^{\left(-\frac{w_{12}\left(c_{1,2}\to 0,\;r,\Omega_{1},\Omega_{2}\right)}{k_{B}T}\right)}-1\right]drd\Omega_{1}d\Omega_{2}.$$

**Figure 1:**
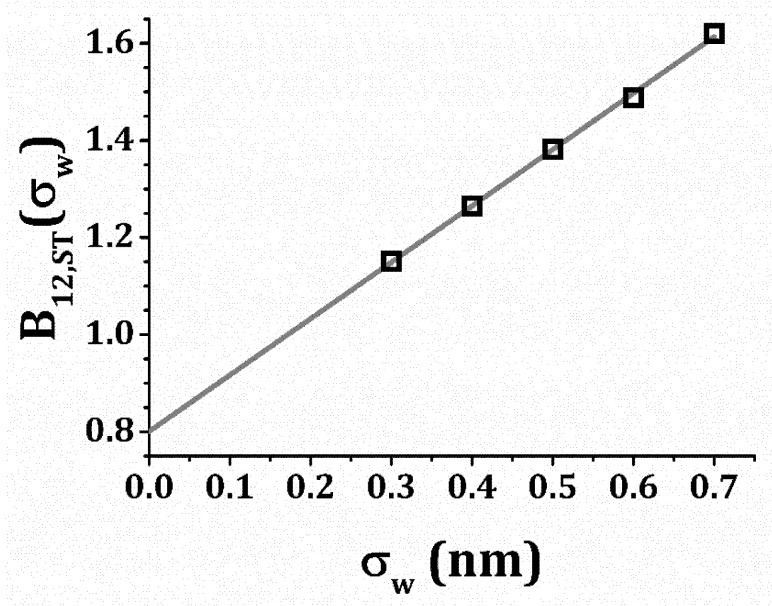
Computed steric contribution to the virial coefficient, *B*_12_, plotted vs. protein hydration layer thickness, *σ_w_*. The points are computed data; the line serves as a visual guide for the extrapolation.

The detailed method of evaluation of Equation (5) is described in Refs. [91,92] Several simulations of *B*_12,*ST*_ as a function were performed for an assumed water hydration layer thickness *σ_w_* in a range, 3 nm ≤ *σ_w_* ≤ 7 nm. A linear extrapolation to the limit *σ_w_* = 0 nm for the mAbs investigated indicates a specific volume estimate, *v*_2_ ≅ 1.14 ± 0.03 mL g^−1^, if *v*_2_ is equated with 2*B*_12,*ST*_ in the *σ_w_* → 0 limit. Figure 1 shows this extrapolation procedure for mAb-1. 

## 3. Results

### 3.1. Biophysical Characterization

#### Size Exclusion Chromatography (SEC)

The ratio of monomer and aggregate content in solution is well appreciated in determining the shear viscosity of protein and antibody solutions, which necessitates the need to make SEC measurements [8,16,93,94]. Therefore, solution SEC data at 25 °C are provided in Table 2. The IgG molecules are mostly in the monomeric state (97.0%) in the mobile phase buffer solution. There is a notable drop in the monomer content for mAb-3 at pH 6.2 to 95.8%. These SEC data will be invoked further during a discussion of the Huggins coefficient data.

### 3.2. Antibody Solution Viscosity and Its Reduction

**Figure 2:**
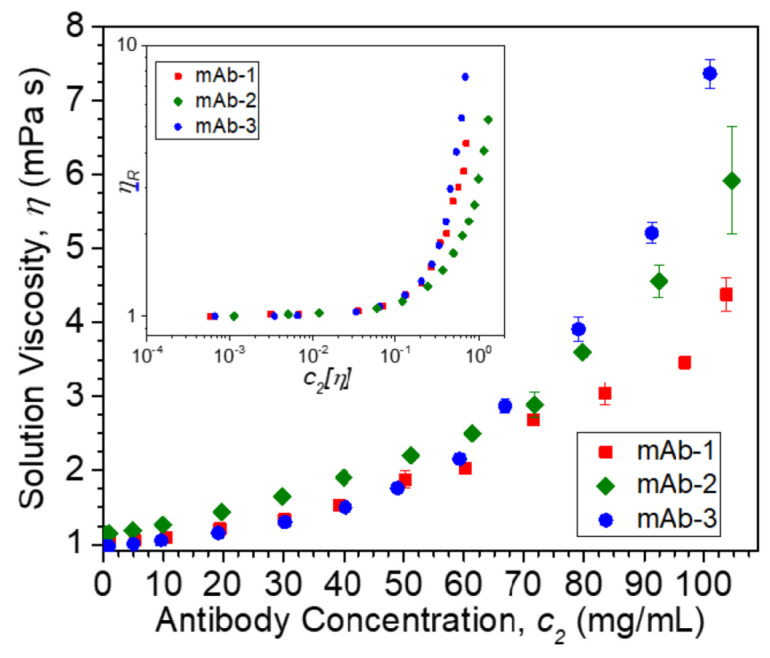
Viscosity *η* at 25 °C vs. mass concentration units (*c*_2_) of mAb formulations. Inset shows relative viscosity *η_R_* plotted vs. dimensionless concentration *c*_2_ [*η*]. Bars on data points denote standard deviations. Note that mAb-2 buffer contains 5% (*w*/*v*) sucrose and 2.5% (*w*/*v*) sorbitol, resulting in higher *η* values than mAb-1 and mAb-3 buffers. For both mAb-1 and mAb-2, each data point was an average of *n* = 3 measurements, while for mAb-3, each data point was an average of *n* = 5 measurements.

**Figure 3:**
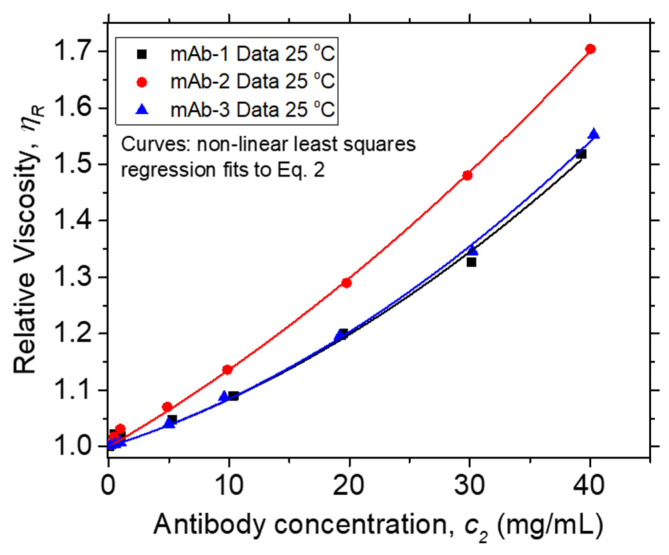
Relative viscosity *η_R_* at 25 °C vs. mass concentration units (*c*_2_) of Vaccine Research Center (VRC) mAbs. Points and curves denote data and non-linear least squares regression fits to Equation (2), respectively. *R*^2^, values for fits to mAb-1, mAb-2, and mAb-3 data are 0.999, 0.992, and 0.999, respectively.

Solution *η* data at 25 °C vs. *c*_2_ are plotted in Figure 2. The inset plot of dimensionless *η_R_* vs. dimensionless *c*_2_ [*η*] in Figure 2 collapses data for different mAbs in their formulation buffers at low protein concentrations onto a single curve, as is commonly observed in polymer solutions under fixed solvent quality conditions [95]. In such an “equation of state data reduction” [96], the solution concentration is conventionally rendered dimensionless using the intrinsic viscosity, which defines a hydrodynamic volume. The breakdown of this reduction occurs near the “overlap concentration”, which is defined by the condition, *c*_2_ [*η*] ≈ 1. The lack of *η_R_* data superposition in this figure when *c*_2_ [*η*] > 0.1 is due to the variable solvent quality of the different antibody formulations, and we then expect *k_H_* to quantify the non-ideality of these inter-protein interactions. The values of *k_H_* and [*η*] extracted from non-linear least squares regression fits of *η_R_* vs. *c*_2_ to Equation (2) (Figure 3) are provided in Table 3. The magnitude of *k_H_* in these mAbs agrees well with values reported by Yadav et al. [97] for other mAbs: 1.5 ≤ *k_H_* ≤ 6.6 and [*η*] ≈ 6 mL/g. Values in this range have been reported in charged biopolymer systems, but these values exceed estimates reported for uncharged flexible polymers [98,99]. This observation provides an important clue that long-range interactions are relevant to understanding the *k_H_* values of these antibody solutions.

Intrinsic viscosity [*η*] measurements are difficult to perform accurately, and there are correspondingly appreciable uncertainties in Huggins coefficient *k_H_* estimates in all studies, even including model systems such as near monodisperse uncharged flexible synthetic polymers in organic solvents [98]. Therefore, we have applied two common methods of estimating *k_H_* to check on the consistency of our estimations under these circumstances–the “Huggins Equation”,

(6) $$\frac{\eta_{sp}}{c}=\left[\eta \right]+k_{H}{\left[\eta \right]}^{2}c$$

And virial expansion (see Equation (2) and non-linear regression fits in Figure 3). The results from both these approaches agree within experimental uncertainties, and this fact provides greater confidence in the numerical estimation of both [*η*] and *k_H_*. The detailed results are provided in the Supporting Data for this manuscript.

**Figure 4:**
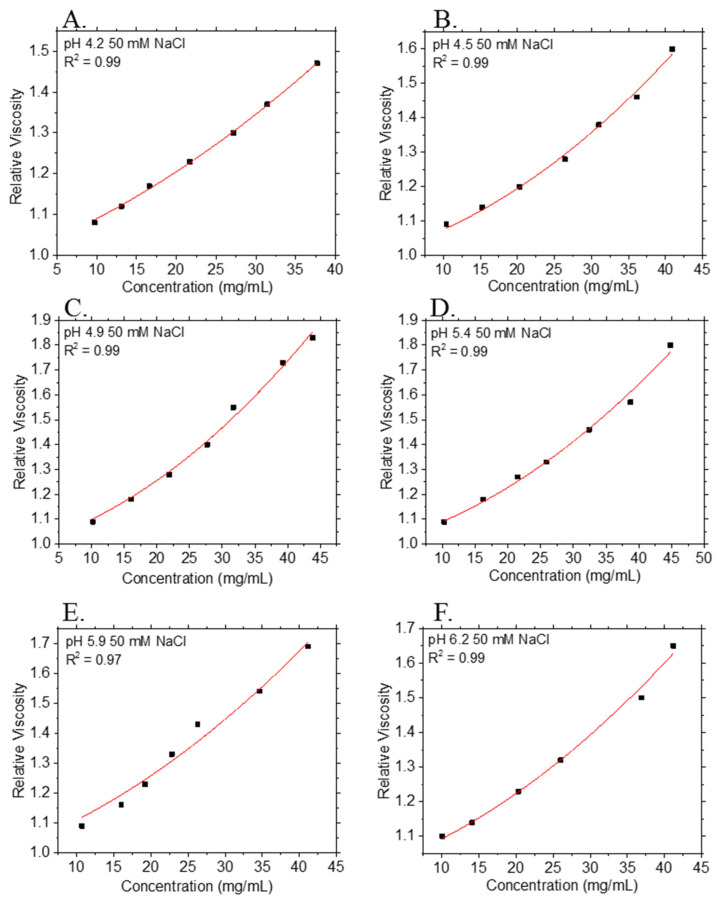
Relative viscosity *η_R_* at 25 °C vs. mass concentration (*c*_2_) of mAb-3 formulation in buffers having a range of pH values: (**A**) pH = 4.2; (**B**) pH = 4.5; (**C**) pH = 4.9; (**D**) pH = 5.4; (**E**) pH = 5.9; (**F**) 6.2. Points and curves denote experimental data and non-linear least squares regression fits to Equation (2), respectively. *R*^2^ values for fits are provided in the legends. Each data point is an average of *n* = 5 measurements, except for data at pH = 5.9: each data point is an average of *n* = 3 measurements. Although no error bars are displayed, all data points have a percentage relative standard deviation of <5.0%.

In addition to measurements of the viscosity of each antibody in its respective formulation buffer, the viscosity of mAb-3 was measured as a function of pH, because pH is an important solution property that sets the net charge on protein molecules and affects their conformation as well as protein–protein interactions. The *η_R_* vs. *c*_2_ data for mAb-3 solutions across the pH range 4.2 ≤ pH ≤ 6.2 and non-linear regression fits to Equation (2) are shown in Figure 4. The histidine-acetate buffering system chosen here allows a precise control of pH over small pH changes across this pH range. The values of *k_H_* and [*η*] extracted from non-linear least squares regression fits of Equation (2) to the data are provided in Table 4.

Unsurprisingly, our *k_H_* estimates on pH provide further evidence of charge interactions in our protein formulations. The isoelectric point (pI) of mAb-3 is ≈ 9.0; as the |pI−pH| increases, the surface charge on the mAb increases, resulting in increased electrostatic repulsion between the protein molecules. As seen in the mAb-3 samples at various solution pH, a lower pH results in *Ψ* values greater than unity. This trend is also expected for objects that that are asymmetric in shape and interact with each other through repulsive interactions. It is clear from Table 4 that [*η*] in volume fraction concentration units is larger than the classical Einstein hard sphere value^30^ of [*η*] = 2.5. For mAb-3, any pH dependence of [*η*] is hard to discern, given the uncertainty in estimation of [*η*] at some of the pH values, even though the correlation coefficient R^2^ is ≥ 0.97 for the fits to the *η_R_* data. Together, these results point to the importance of particle shape on these virial coefficients and the limitations of the idealized hard sphere model of proteins that pervades the literature. These conclusions are qualitatively in accord with the messages from a previous paper by a subset of the authors [14,102].

## 4. Discussion

### Comparison of Antibody Solution Measurements with Flexible Polymer and Sticky Sphere K_h_ Models

To better motivate a discussion of Huggins coefficient in terms of idealized colloidal and polymer models of antibodies and the consequent comparisons to molecular theories, a review of the definitions for conventional concentration units used in polymer and colloid science and issues related to the inter-conversion between these units is warranted. In polymer and protein science, it is conventional to measure the macromolecule solute concentrations *c*_2_ in units of g of macromolecule/mL solution. Then, the solution viscosity *η* is described by the same power series expansion as in Equation (2).

*η_R_* = *η*/*η_s_* = 1 + [*η*] *c*_2_ + *k_H_* ([*η*] *c*_2_)^2^ + … (7)

Here, [*η*] correspondingly has units of mL/g, because *η_R_* is dimensionless, by definition. It is also traditional in polymer science to introduce reduced concentrations defined in terms of a polymer “overlap concentration” [89] c*, at which inter-polymer interactions start to become appreciable. Specifically, Equation (7) is formally re-written as,

*η*/*η_s_* = 1 + (*c*_2_/c*) + *k_H_* (*c*_2_/c*)^2^ + *O*(*c*_2_/c*)^3^, (8)

where the overlap concentration that signifies the onset of inter-polymer interaction is defined as c* = 1/[*η*]. The chain radius of gyration *R_g_* may also be used to define dimensionless concentration units for polymer solutions based on the concept of geometrical overlap [103,104], but dynamical properties such as *η* are usually defined in terms of [*η*]. Note that *k_H_* is theoretically invariant to the choice of concentration units, although the conversion between mass concentration units to volume fraction units can give rise to uncertainties associated with estimating the protein molecular volume. As will be discussed below, *k_H_* in polymers typically ranges from about 0.7 in a good solvent where excluded volume repulsions are strong, as in solutions of hard spheres, to a value near 0.3 in a *θ* solvent where attractive interparticle interactions between the polymers exactly balance the repulsive interactions in *B*_22_, i.e., *B*_22_ (*T* = *T_θ_*) = 0 [13]. This “ideal” or “*θ* point” state for solutions of polymers or particles at which interparticle excluded volume interactions effectively vanish is the exact analog of the Boyle temperature, *T_B_*, in non-ideal gases [13,105].

The viscosity virial expansion for suspensions of hard sphere Brownian particles is naturally described in terms of a virial expansion in terms of volume fraction [106,107].

*η*/*η_s_* = 1 + 2.5 *ϕ*_2_ + 6.2 *ϕ_2_*^2^ + *O*(*ϕ*_2_^3^) = 1 + 2.5 *ϕ*_2_ + *k_H_* (2.5 *ϕ*_2_)^2^ + *O*(*ϕ*_2_^3^) (9)

The leading term 2.5 pre-factor is due to Einstein [108] and the second-order viscosity virial coefficient 6.2 is due to Batchelor^14^. The “*O*” symbol denotes “order of magnitude”. As noted before, *k_H_* for the sticky hard sphere model, where there is a variable short-range attractive interaction, is given by Equation (4). Then, we may deduce the repulsive hard sphere estimate for the Huggins coefficient, *k_H_* ≈ 0.99 ≈ 1.0, by inserting *Ψ* = 1 in Equation (4).

Then, these colloidal estimates can be compared with the theoretical estimate of *k_H_* for flexible polymer solutions in a *θ* solvent, *k_H_* (*Ψ* = 0) ≈ 0.757 derived by Edwards and Freed [109] and the good solvent estimate *k_H_* (*Ψ* = 1) *≈* 0.4 of Muthukumar and Freed [110]. Yamakawa [111] derived an expression for *k_H_* covering intermediate degrees of excluded volume interaction strength, 0 < *Ψ* < 1, which can be approximately expressed as *k_H_* ≈ ½ − 0.3 *Ψ*, corresponding to a variation between 0.5 and 0.2 upon going from *θ* solvent to a good solvent; the variation is nearly linear with the *B*_22_, as suggested by the sticky sphere model variation of *k_H_*. Classical measurements by Berry [99] on polystyrene solutions support these theoretical predictions to a reasonable approximation, although the experimental estimates have tended to be a little smaller in magnitude than the theoretical predictions for *k_H_*. Evidently, *k_H_* for polymer solutions has a similar qualitative variation with “solvent quality” (*Ψ*) as for the stick sphere model, but the magnitude of *k_H_* is significantly larger for hard sphere particles. This qualitative difference in the relative magnitude of *k_H_* offers a chance to gauge whether antibodies resemble spherical colloidal particles or flexible polymers, and we next consider an estimate of *k_H_* for our antibody solutions over a broad range of solvent quality.

The observation noted above brings us to a consideration of whether *B*_22_ measurements provide valuable insight into the change in *η_R_* exhibited in Figure 3 and Figure 4. *B*_22_ values of our mAb solutions were determined using SLS, and they were normalized by *B*_22,*ST*_ to calculate *Ψ*. Grünberger et al. [100] and Calero-Rubio et al. [91,101] have calculated *B*_22,*ST*_ for IgG1 mAbs: *B*_22,*ST*_ *≅* 10 mL g^−1^. The *Ψ* values for these mAbs (Table 3 and Table 4) are ≅ 0.5–0.7, in contrast to NIST mAb, for which *Ψ* = 1.99 (NIST mAb *B*_22_ = 19.9 mL/g; personal communication from Vincent Shen and Marco Blanco-Medina, NIST, Gaithersburg, MD, USA) (If proteins were true hard spheres, then *Ψ* could not exceed 0.99, providing further evidence that antibodies cannot be described physically as hard spheres).

We obtain further insight into this situation through a consideration of recent osmotic pressure and scattering measurements of serum albumin solutions [112], where the properties of their protein solution were found to be consistent with a solution of oblate ellipsoids. If we accept this as being an acceptable “coarse-grained” model of our Y-shaped antibodies with a rough estimate of aspect ratio (5 × 1), then *Ψ* can be exactly computed to equal, *Ψ* (hard oblate ellipsoid; 1 × 5) = 1.89 [113].

**Figure 5:**
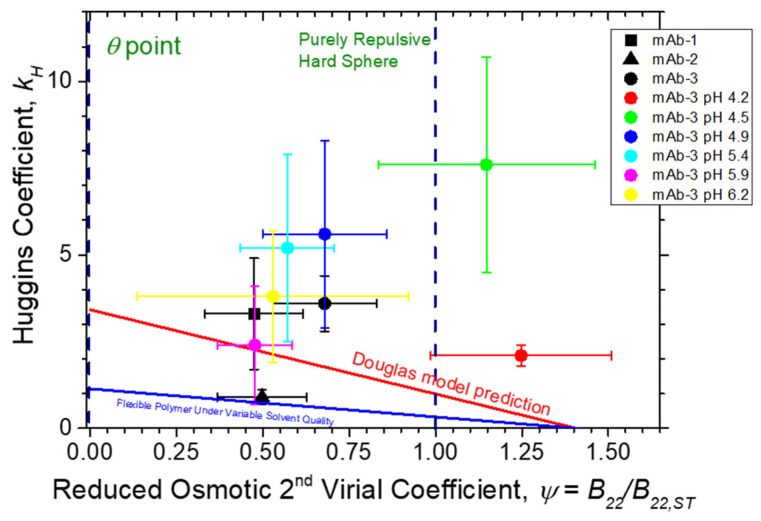
Huggins coefficient *k_H_* vs. reduced osmotic second virial coefficient. Comparison of *Ψ* (circles) to Douglas et al. [23] model prediction (continuous red line; Equation (4)) and an estimate (see text for discussion) for flexible polymer chains with variable excluded volume interaction (continuous blue line). Uncertainty estimates were determined from the non-linear regression of Equation (2) to *η* data, and linear regression of Equation (6) to SLS *Kc*_2_/*R_θ_* data. Dashed vertical lines denote limiting *k_H_* values at the *θ* point (*Ψ* = 0) and for purely repulsive hard spheres (*Ψ* ≈ 0.99), respectively.

If we simply ignore the obvious difficulty of treating antibodies as being hard sphere and plot *k_H_* as a function of *Ψ*, then we may check whether at least the qualitative trends of sticky hard sphere and polymer models are followed. In Figure 5, we directly compare our antibody data for *k_H._* vs. *Ψ*. Our observations indicate that there might be a general trend that *k_H_* increases as ψ is reduced, but there is clearly no quantitative agreement with either the sticky hard spheres or polymer with variable excluded volume interaction models. Data for mAb-2 do approach the flexible polymer chain prediction, but the data for mAb-1 and mAb-3 clearly depart from the flexible polymer chain model. The pH-dependent data for mAb-3 do not even qualitatively agree with the flexible polymer chain model and the data for mAb-1, mab-2, and mAb-3 are also not well described by the adhesive hard sphere model estimate of *k_H_* in terms of ψ introduced by Douglas et al. [23]. Meanwhile, the pH-dependent data for mAb-3 admittedly have appreciable uncertainty. Pamies et al. [98] have noted the challenges associated with the determination of [*η*] and *k_H_* by single-point and dilution procedures, and the large uncertainty associated with the determination of *k_H_*; their lack of agreement with both the hard sphere and the flexible polymer chain models is an effect beyond this uncertainty. We note that the antibody solution model of Kastelic et al. [71] mentioned above also predicts that *k_H_* varies linearly with *Ψ*, but the predicted variation of *k_H_* appears to be much weaker than the colloid sphere model of Russel. In particular, Kastelic et al. find *k_H_* to equal 0.6 near the theta point where *Ψ* vanishes, which is in the range (0.5, 0.57) of the estimates of *k_H_* for the polymer model.

This deviation is understandable given the lack of consideration of charge, dipole interactions, hydration, and other factors known to contribute to protein–protein interactions in protein solutions. As seen in both Table 4 and Figure 5, under acidic pH conditions, *Ψ* for mab-3 is significantly larger than its theoretically maximum value for a repulsive hard sphere, i.e., *Ψ* = 1.0, which might be attributed to an increase in repulsive electrostatic interactions or perhaps some deviation from a spherical protein shape based on a simple colloidal sphere protein. However, the increasing trend in *Ψ* with lowering pH is not reflected in changes in the intrinsic viscosity values, which instead exhibit a minimum at pH 4.5. A simple change of protein shape simply cannot reasonably be invoked to rationalize the large value of *k_H_* observed in our measurements. Accordingly, we interpret the large value of *k_H_* to arise from a large dipole–dipole interaction, as discussed above [31,34,56,97,114]. Of course, charge and dipolar interaction effects should also make a significant contribution [115] to $B_{22}$, but even under conditions of strongly repulsive protein–protein interactions, we still see a deviation from the colloidal sphere model, which would generally require *Ψ* < 1. We concur with other recent studies of antibody solutions noted above indicating the complete inadequacy of the hard sphere model of the solution viscosity and other solution properties of antibody protein solutions, including our past study coming to the same conclusion [14]. We must add to this conceptual pyre [102] the neutral polymer model of antibodies, which is also a clearly physically inadequate model of this class of proteins. Clearly, an improved understanding of protein hydration, ion solvation, and dipole–dipole, charge–dipole, and perhaps multipole interactions is required to fully understand the molecular significance of the viscometric interaction parameter *k_H_* of antibody solutions. Despite these difficulties, *k_H_* along with *k_D_* [26,27] provides a useful practical measure of the strength of inter-protein interactions at elevated protein concentrations of direct significance to developing antibody formulations that minimize the solution viscosity for many clinical applications.

## 5. Conclusions

This work has provided the experimental measurements of Huggins coefficient, *k_H_*, a classical measure of the interparticle interaction strength, for three monoclonal antibodies in solution. The intrinsic viscosity [*η*] of these antibody solutions, a basic measure of macromolecular shape/conformation, was determined by using the relative viscosity data from classical capillary viscometry. The range of 6.0 ≤ [*η*] ≤ 10.0 for these antibodies significantly exceeds the classical Einstein result of 2.5 for hard spheres, confirming that these antibodies do not behave as hard spheres. Another basic measure of inter-protein interaction, the second osmotic virial coefficient, *B*_22_, was also measured using static light scattering with the objective of critically scrutinizing literature-proposed models of proteins, viz., the sticky hard sphere model and the flexible chain polymer model. The relative viscosity data for all the antibody solutions tested were reduced to a single curve by use of a dimensionless concentration variable, which is defined as the product of the protein concentration (*c*_2_) and intrinsic viscosity, [*η*]. This successful reduction of viscosity for a range of *c*_2_ [*η*] ≤ 0.1 lends some support for the polymeric nature of antibodies in solution, as solutions of synthetic polymers show similar reduction. However, while there is agreement of the trend in the reported *k_H_* data with the magnitude of *B*_22_, these measurements clearly indicate that these antibodies cannot be quantitatively modeled as either simple hard spheres or as flexible uncharged polymers. Instead, these measurements point to the existence of significant attractive interactions between the antibody molecules that cause the viscosity to increase more rapidly than in the case where the protein molecules interact with each other by short-range interactions only. We suggest that these interactions derive from solvation and counter-ion association of these charged polymers, and potentially due to dipole–dipole interactions, and that these interactions need to be studied in depth in order to understand the molecular origin of the values of *k_H_* that are reported. Nonetheless, *k_H_* provides a valuable measure of protein–protein interaction in relation to the increase of solution viscosity, and this quantity should be useful in the design of protein formulations that minimize the increase of viscosity for a given protein therapeutic concentration. Developing a better molecular understanding of the interactions underlying *k_H_* will be helpful in designing stable antibody formulations for clinical trials of therapeutics and vaccines, whose viscosity is tractable for self-administration device design.

To paraphrase Einstein’s general comment on the development of models: “Everything should be made as simple as possible, but no simpler” [116].

## Figures and Tables

**Table 1:** Monoclonal antibodies (mAbs) and formulation buffer compositions.

Antibody	PDB ID	Molar Mass (*kDa*)	Protein Iso-Electric Point Main Peak/Range	Buffer Composition	Net Ionic Strength (mM)
mAb-1	4LST	148	9.06; 8.78–9.25	25 mM Na citrate, 50 mM NaCl,150 mM arginine HCl, pH 5.8	278.8 *
mAb-2	5FYJ	151	9.30; 9.14–9.59	50 mM histidine HCl, 50 mM NaCl, 5 % (*w*/*v*) sucrose, 2.5% (*w*/*v*) sorbitol, pH 6.8	56.9
mAb-3	5TE4	158	9.13; 8.99–9.47	10 mM Na citrate, 50 mM NaCl 150 mM arginine HCl, 0.002% (*w*/*v*) polysorbate 80, pH 6.5	246.3
mAb-3 (pH Variation)	5TE4	158	-	20 mM histidine acetate, 50 mM NaCl 4.2 ≤ pH ≤ 6.2	70 †

* Assuming arginine hydrochloride is in +1 state, which should be accurate. † 20 mM histidine-acetate buffer was prepared by mixing the pure solid histidine base with liquid acetic acid, and the ionic strength from the buffer is always 20 mM, since no other counterions were introduced into the system. Detailed calculations of ionic strength are provided in Supporting Information.

**Table 2:** Size exclusion chromatography (SEC) data on monoclonal antibodies.

System	% Monomer	% Fragment	% Aggregate
mAb-1 formulation	99.0	0.0	1.0
mAb-2 formulation	98.0	0.0	2.0
mAb-3 formulation	99.0	0.0	1.0
mAb-3 pH 4.2	98.8	0.4	0.8
mAb-3 pH 4.5	98.6	0.3	1.1
mAb-3 pH 4.9	97.8	0.2	2.0
mAb-3 pH 5.4	97.8	0.0	2.2
mAb-3 pH 5.9	97.1	0.0	2.9
mAb-3 pH 6.2	95.8	0.0	4.2

**Table 3:** Experimentally determined values of Huggins coefficient *k_H_*, intrinsic viscosity [*η*], and osmotic second virial coefficient *B*_22_ at 25 °C for mAbs.

Antibody in Buffered Formulation	Huggins Coefficient, *k_H_* ^2,3,4^	Intrinsic Viscosity [*η*], mL/g ^1,2,4^	[*η*] Volume Fraction Units	*Ψ* ≡ *B*_22_/*B*_22,_*_ST_* ^1,5^
mAb-1	3.3 ± 1.6	6.8 ± 1.0	6.0 ± 0.9	0.47 ± 0.14
mAb-2	0.9 ± 0.2	12.4 ± 0.7	10.9 ± 0.6	0.50 ± 0.13
mAb-3 (at fixed pH 6.5)	3.6 ± 0.8	6.8 ± 0.5	6.0 ± 0.4	0.68 ± 0.15

^1^*B*_22_ was normalized as *B*_22_/*B*_22,*ST*_, and [*η*] was made dimensionless by dividing it by *v*_2_. ^2^ *k_H_* and [*η*] were determined from fits of Equation (2) to *η_R_* data limited to *ϕ*_2_ ≤ 0.06, where the virial expansion remains valid. ^3^ Note that *k_H_* is dimensionless in reduced *c*_2_ [*η*] units. ^4^ The uncertainty (±) in *k_H_* and [*η*] was extracted from the non-linear least squares regression fits based on the Levenberg–Marquardt algorithm. ^5^ *B*_22,*ST*_ values calculated for immunoglobulin 1 (IgG1) mAbs were taken from Grünberger et al. [100] and Calero-Rubio et al. [91,101].

**Table 4:** Huggins coefficient *k_H_*, intrinsic viscosity [*η*], and the normalized osmotic second virial coefficient *B*_22_, i.e., *M* *B*_22_/*B*_22,*ST*_ [*η*], at 25 °C for mAb-3.

mAb-3 Solution pH	Huggins Coefficient, *k_H_* ^1–3^	Intrinsic Viscosity [*η*] (mL/g) ^1,3^	[*η*] Volume Fraction Units	*Ψ* = *B*_22_/*B*_22,*ST*_
4.2	2.1 ± 0.3	7.7 ± 0.3	6.8 ± 0.3	1.25 ± 0.26
4.5	7.6 ± 3.1	5.4 ± 0.8	4.7 ± 0.7	1.15 ± 0.31
4.9	5.6 ± 2.7	7.1 ± 1.3	6.2 ± 1.1	0.68 ± 0.18
5.4	5.2 ± 2.7	6.7 ± 1.2	5.9 ± 1.0	0.57 ± 0.14
5.9	2.4 ± 1.7	9.0 ± 1.9	7.9 ± 1.7	0.48 ± 0.11
6.2	3.8 ± 1.9	7.2 ± 1.2	6.3 ± 1.1	0.53 ± 0.50

^1^ Both *k_H_* and [*η*] were determined from fits of Equation (2) to *η_R_* data for the concentration range *ϕ*_2_ ≤ 0.06, where the virial expansion is valid. ^2^ Note that *k_H_* is dimensionless in reduced *c*_2_ [*η*] units. ^3^ The uncertainty (±) in *k_H_* and [*η*] was extracted from the non-linear least squares regression fits based on the Levenberg–Marquardt algorithm.

## Data Availability

The data presented in this study are available on request from the corresponding author.
